# Evaluating Virtual and Inpatient Pulmonary Rehabilitation Programs for Patients with COPD

**DOI:** 10.3390/jpm12111764

**Published:** 2022-10-25

**Authors:** Paula Irina Barata, Alexandru Florian Crisan, Adelina Maritescu, Rodica Anamaria Negrean, Ovidiu Rosca, Felix Bratosin, Cosmin Citu, Cristian Oancea

**Affiliations:** 1 Center for Research and Innovation in Precision Medicine of Respiratory Diseases, “Victor Babes” University of Medicine and Pharmacy Timisoara, Eftimie Murgu Square 2, 300041 Timisoara, Romania; 2 Faculty of Medicine and Pharmacy, University of Oradea, 410073 Oradea, Romania; 3 Department XIII, Discipline of Infectious Diseases, “Victor Babes” University of Medicine and Pharmacy Timisoara, Eftimie Murgu Square 2, 300041 Timisoara, Romania; 4 Methodological and Infectious Diseases Research Center, Department of Infectious Diseases, “Victor Babes” University of Medicine and Pharmacy Timisoara, Eftimie Murgu Square 2, 300041 Timisoara, Romania; 5 Department of Obstetrics and Gynecology, “Victor Babes” University of Medicine and Pharmacy Timisoara, Eftimie Murgu Square 2, 300041 Timisoara, Romania

**Keywords:** COPD, pulmonary rehabilitation, digitalization of healthcare, respiratory disease

## Abstract

Chronic obstructive pulmonary disease (COPD) is an increasingly frequent disorder that is likely to become the third leading cause of morbidity worldwide. It significantly degrades the quality of life of patients affected and poses a significant financial burden to the healthcare systems providing treatment and rehabilitation. Consequently, our study’s purpose was to compare conventional inpatient pulmonary rehabilitation (PR) with virtual (online) PR using a mobile phone application. During a three-month period, two groups of patients followed the research protocol by participating in a pulmonary rehabilitation program administered and supervised by a physical therapist five times per week. A number of respiratory variables were examined before and after the test. At the end of the study period, a total of 72 patients completed the rehabilitation in the inpatient group, respectively 58 in the online group. It was observed that post-test comparison between patients undergoing the traditional and online rehabilitation methods did not show any significant differences. However, the calculated mean differences between pre-test and post-test results were significantly higher in favor of the virtual method. The most significant variations were encountered in maximal inspiratory pressure (MIP) (6.6% vs. 8.5%, *p*-value < 0.001), 6-min walking test (6MWT) (6.7% vs. 9.4%, *p*-value < 0.001), and COPD assessment test (CAT) values (4.8 vs. 6.2, *p*-value < 0.001), respectively. However, the maximal expiratory pressure (MEP) variation was significantly higher in patients undergoing the traditional rehabilitation method, from an average of 4.1% to 3.2% (*p*-value < 0.001). In this preliminary study, the online pulmonary rehabilitation program proved non-inferiority to the traditional method, with significantly better results in several measurements. Additional studies using larger cohorts of patients and longer exposure to the online rehabilitation program are required to validate these findings.

## 1. Introduction

Chronic obstructive pulmonary disease (COPD) is a major cause of chronic morbidity and mortality in the world and is the third leading cause of global disability-adjusted life-years (DALY) [1]. COPD is a progressive respiratory disease that leads to physical inactivity, worsening dyspnoea, muscle deconditioning, and reduced quality of life [2,3].

Although there have been remarkable advances in pharmacological treatments, a large proportion of patients remain symptomatic. Pulmonary rehabilitation (PR) has been recognized as an important, standard treatment for people with COPD aimed at reducing the burden of symptoms by increasing exercise tolerance and improving self-management. The provision of PR is mandated by the National Institute for Health and Care Excellence (NICE) as a key pillar of integrated care [3].

There is level 1 evidence that PR improves dyspnoea, exercise capacity, and quality of life, regardless of disease severity [4]. Despite these findings, 5% of people who would benefit from PR undertake it [5] with a low referral (<15%) [6], high non-attendance (up to 50%), and poor completion rates (up to 30%) [7].

Moreover, approximately 50% of patients with severe and very severe COPD declined to participate in these programs, and between 30–50% dropped out before completion [8]. The barriers to the uptake of a PR program include lack of transportation, perceived benefits of PR, disruption of the usual routine, the timing of programs, lack of rehabilitation centers, and shortage of qualified health professionals [7].

Since 2015, the American Thoracic Society (ATS) and European Respiratory Society (ERS) have recommended investigating alternative approaches to PR in an attempt to increase uptake and make PR available to more patients [9]. Home-based models of PR have been proposed to increase the availability and accessibility of PR programs to patients [10], moreover during the COVID-19 pandemic to facilitate social distancing. Although telerehabilitation has existed for many years, this model’s clinical efficacy is still unclear. Therefore, the objective of our study was to compare traditional inpatient PR with online PR through a mobile phone application.

## 2. Materials and Methods

### 2.1. Study Design

The patients were recruited from the Pneumocontrol application database. These were the patients who, during the SARS-CoV-2 pandemic, accessed the application for pulmonary rehabilitation information. The patients were randomly selected, and two groups were formed. The first group was the inpatient group that received a conventional pulmonary rehabilitation program, and the second group was the online group that performed PR through the application. The study was conducted over a period of three months, from January 2022 to April 2022.

All the patients were informed of the research, and informed consent was obtained before the beginning of the study. The research respected the Declaration of Helsinki regarding ethical principles for research regarding the safety of human subjects. The study design and contract forms were approved by the Ethics Committee of the “Victor Babes” Hospital (nr.3209 5 April 2022).

Both groups had to perform one pulmonary rehabilitation program conducted and supervised by a physical therapist five times a week. The duration of the program was 21 days. At the beginning and at the end of the program, all patients performed: lung volumes, maximal inspiratory and expiratory pressure (MIP/MEP), 6-min walking test, COPD assessment test (CAT), Borg scale, and modified Medical Research Council test (mMRC).

Patients in the online group were explained how to use the application, exercise with the POWERbreathe device, and how to increase weight through the sessions.

### 2.2. Patients

Over a period of three months, we included patients with stable COPD that were classified according to the ATS/ERS criteria for the severity of airway obstruction [11]. Inclusion criteria: age > 45 years, will participate, no exacerbation in the last three months, no prior rehabilitation in the last three months, former smoking history, non-smoking status, owning a mobile smartphone, able to use a smartphone, stationary bicycle at home (for the online group), owning a pulse oximeter.

Exclusion criteria: exacerbation in the last three months, other comorbidities that could interfere with their current health status, use of medication that could affect exercise response, active smoking status, musculoskeletal conditions that could impair exercise, an impaired vision that could affect the use of the mobile application, not having a stationary bicycle at home, a cognitive impairment that could affect the understanding of the exercises. After applying the inclusion and exclusion criteria, there were 72 patients in the inpatient group and 58 patients in the online group.

### 2.3. Lung Volumes and Respiratory Strength

The lung volumes and respiratory muscle strength were determined using the Smart PFY UI device (medical equipment Europe GmbH). The patients were seated in an upright position with the feet flat on the ground and performed three maximal expirations. The best value was recorded. The inclusion criteria for the patients were according to the ATS/ERS guideline using the refined ABCD assessment tool [11,12]. To determine respiratory muscle strength, three assessments were recorded, and the best value was used. All the maneuvers were performed according to standard procedures [13].

We used the same device as for spirometry but adapted with a shutter module. To determine maximal inspiratory pressure, the patients were instructed to expire to residual volume followed by a maximum inspiration against a resistance applied by the module. Three expirations were performed, and the best values was recorded. Maximal expiratory pressure was assessed by instructing the patient to breathe into total lung capacity, followed by a forced expiration against the module.

### 2.4. Physical Capacity, Disease Impact, and Dyspnea

Physical capacity was assessed using the 6-min walking distance test (6MWD), in which the patient had to cover as much distance as possible in the predicted time. To perform the 6MWD we used the ERS/ATS recording form, the BORG scale, pulse-oximeter and a stopwatch. The test was performed on a 30 m long corridor according to the ATS guidelines [14].

The global impact of COPD on the patient was evaluated using the COPD assessment test (CAT questionnaire). The questionnaire consists of 8 questions on a numerical scale from 0 to 5 for each question. Higher scores denote a more severe impact of COPD on a patient’s life [15].

We assessed dyspnoea with the Borg breathlessness scale, which rates the difficulty of breathing. It rates the breathing on a scale from 0 to 10, where 0 means “breathing causes no difficulty” and 10, where “breathing is maximal”. We also used this scale to determine the effort level during training sessions [16].

Dyspnoea was also evaluated using the modified Medical Research Council scale (mMRC), which assesses the degree of baseline functional disability due to dyspnoea. It rates dyspnoea on a scale from 0-“dyspnoea only with strenuous exercise” to 4-“too dyspneic to leave the house or breathless when dressing” [17].

### 2.5. Intervention

Inpatient pulmonary rehabilitation was performed with a physical therapist in the hospital. Home monitoring and training exercises were performed online through the Pneumocontrol application. The feasibility of the application was demonstrated in previous studies [18,19]. Patients were given basic instructions on how to use the online application after making sure the internet connection was working, and a brief test was performed before first use in live session with one of the researchers involved in the study. The training sessions lasted 45–60 min and included diaphragmatic breathing, pursed lips breathing, and strength and endurance training for both upper and lower extremities, according to the recommendation of the American Thoracic Society [20].

Each training session was composed of: (a) warm-up: duration 5–10 min—sitting, standing warm-up exercises, different breathing techniques; (b) endurance training: duration 20–30 min—stationary bicycle, Borg between 5–7, exercise performed continuous or intervals; strength/resistance training: duration 20–30 min—50–80% of 1 RM, 10–15 repetitions, three sets; cool-down: duration 5–10 min—stretching, different breathing techniques.

Respiratory muscle training was performed once per day before the training session with the POWERbreathe MEDIC device. Patients had to inhale through a variable-diameter orifice. The smaller the orifice, the greater the load achieved. Thirty breaths had to be performed per session at a level that was determined for each patient.

All the exercises were performed respecting basic physical education principles. Patients started with light and simple exercises, and as they progressed, the exercises became more complex. All training sessions were individualized according to the patients’ possibilities, scores, and symptoms obtained from the questionnaire in the application.

### 2.6. Statistical Analysis

Microsoft Excel and IBM SPSS (Armonk, NY, USA: IBM Corp.) were the programs used for statistical analysis. The presentation of continuous variables included the use of the mean and standard deviation (SD) if the variable followed a Gaussian distribution (Kolmogorov–Smirnov test). In order to determine the difference between the normally distributed variables, the Student’s *t*-test was used in order to provide an estimate of the *p*-value. The Chi-square and Fisher’s tests were carried out to investigate the proportional differences. A Mann–Whitney U-test was performed for the mMRC scale to compare the mean ranks. It was decided that a *p*-value of 0.05 was significant for statistical analysis.

## 3. Results

### 3.1. Comparison of Pre-Test and Post-Test Results in the Inpatient Setting

**Figure 1:**
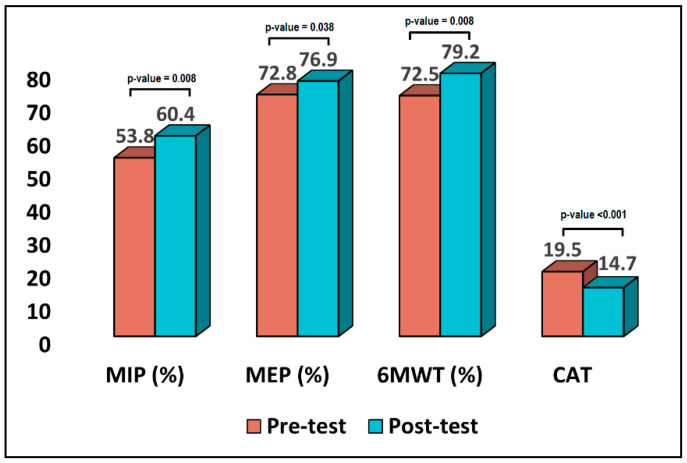
Comparison of pre-test and post-test results in the inpatient setting.

The current study enrolled a total of 72 patients in the inpatient setting and 58 in the online setting. The variables of interest from both study groups were measured before and after the intervention. As presented in Table 1, the average patient age in the inpatient setting was 64.9 years, while most of them were men (75.0%), with an average BMI of 25.4 kg/m^2^. Regarding pulmonary parameters, the predicted FVC value was 4.1 L, with no significant difference in actual and (%) values. Similarly, the FEV1 predicted value was 3.0 L, with no significant difference in actual and (%) values. The MIP (%) and MEP (%) comparison between pre- and post-test results showed a difference from 53.8% to 60.8% (*p*-value = 0.006), respectively, from 72.8% to 76.9% (*p*-value = 0.038), the difference between these measurements being statistically significant, as observed in Figure 1. The CAT measurement was 19.5 before intervention and 14.7 after intervention (*p*-value ≤ 0.001). Lastly, the mMRC results also showed a statistically significant decrease from a mean rank of 45.25 pre-test to 27.75 post-test (*p*-value ≤ 0.001).

### 3.2. Comparison of Pre-Test and Post-Test Results in the Online Setting

**Figure 2:**
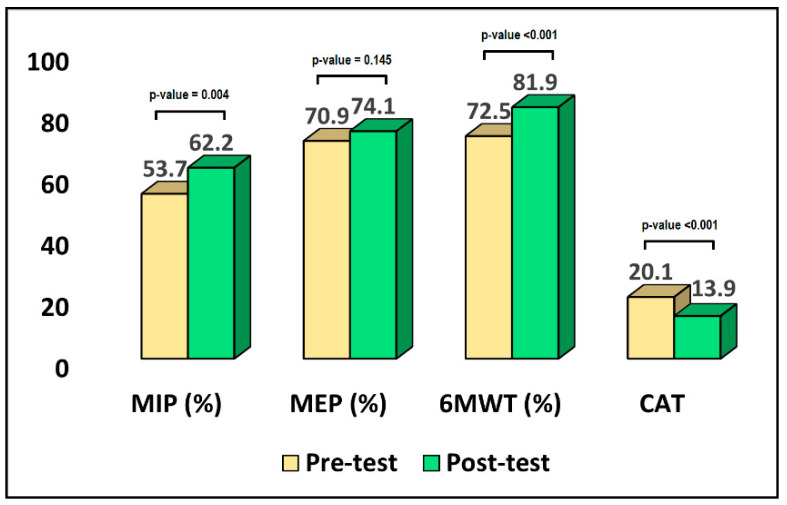
Comparison of pre-test and post-test results in the online setting.

As described in Table 2, the average patient age in the inpatient setting was 64.3 years, while most of them were men (72.4%), with an average BMI of 25.7 kg/m^2^. Regarding pulmonary parameters, the predicted FVC value was 4.1 L, with no significant difference in actual and (%) values. Similarly, the predicted FEV1 value was 3.0 L, with no significant difference in actual and (%) values. The MIP (%) and MEP (%) comparison between pre- and post-test results showed a difference from 53.7% to 62.2% (*p*-value = 0.004), respectively, from an average of 70.9% to 74.1% (*p*-value = 0.145), as seen in Figure 2. The 6MWT levels were statistically significantly different between the pre-test and post-test measurement (342.9 vs. 387.2, *p*-value = 0.006). The CAT measurement was 20.1 before intervention and 13.9 after intervention (*p*-value < 0.001). Lastly, the mMRC results showed a statistically significant decrease from a mean rank of 39.48 pre-test to 19.52 post-test (*p*-value = 0.004).

### 3.3. Comparison of Pre-Test Results between Inpatients and Online Participants

The pre-test comparison between inpatients and online participants presented in Table 3 identified no statistically significant differences between the two study groups, providing an excellent basis for the post-test analysis by removing any suspicion that future changes might be caused by initial differences between groups. The actual FVC value in the inpatient group before intervention was 2.9 L compared with 3.0 L in the online group (*p*-value = 0.105). Similarly, the actual FEV1 value was 1.3 L in the inpatient group, compared with 1.4 L in the online group (*p*-value = 0.066). The comparison of mMRC pre-test results between patients in the inpatient and online settings did not show significant differences in mean ranks (33.71 vs. 32.12, *p*-value = 0.696).

### 3.4. Comparison in Post-Test Results between Inpatients and Online Participants

Comparable to the pre-test measurements, the post-test measurements presented in Table 4 discovered statistically significant differences between inpatient and online participants with regard to the actual FVC and FEV1 levels. The FVC in the inpatient group was 2.9 L compared with 3.1 L (*p*-value = 0.004), and the actual FEV1 in the inpatient group was 1.2 L compared with 1.4 L (*p*-value = 0.010), respectively. The comparison of mMRC pre-test results between patients in the inpatient and online settings did not show significant differences in mean ranks (35.33 vs. 30.10, *p*-value = 0.222). Although the actual measured post-test results between patients undergoing the traditional and online rehabilitation methods did not show many significant differences, the calculated mean differences between pre-test and post-test results were significantly higher in favor of the online method, as seen in Table 5. Therefore, the most significant variations were encountered in MIP (6.6% vs. 8.5%, *p*-value < 0.001), 6MWT (6.7% vs. 9.4%, *p*-value < 0.001), CAT values (4.8 vs. 6.2, *p*-value < 0.001), respectively. However, the MEP (%) variation was significantly higher in patients undergoing the traditional rehabilitation method (4.1% vs. 3.2%, *p*-value < 0.001).

## 4. Discussion

### 4.1. Important Findings

The lockdown during the COVID-19 pandemic had a major effect on patients with respiratory diseases, who were no longer able to access respiratory rehabilitation services with the same ease [21]. Moreover, due to the imposed governmental restrictions, the level of physical activity of these patients also suffered, since everyone was advised to stay at home. Considering these conditions, respiratory rehabilitation programs had no other option but to move to the online environment or to different mobile phone platforms.

Digitalized respiratory rehabilitation performed online or through various platforms is not new, having its birth in 2008, when Liu et al. tried using the mobile phone to provide exercises that improve walking exercise [22]. The same objective was followed by the current study in order to compare respiratory rehabilitation in hospitalized patients with online respiratory rehabilitation using a mobile phone application.

When we compared each group separately, we noticed that all patients showed significant improvements in all studied parameters. When comparing both groups, we observed that there were no significant differences after 21 days of pulmonary rehabilitation. Our findings support the results of other studies and the hypothesis that there is no difference between these two approaches to delivering pulmonary rehabilitation and that both can improve outcomes, in association with smoking cessation [23].

Compared to other studies that used online rehabilitation [22,24], interactive web-based applications [25], supervised telerehabilitation [26], and home-based telerehabilitation using video conference, we used an application on a mobile phone. The feasibility and utility of this application were previously demonstrated in other studies [18,19].

To our knowledge, this is the first study that, besides the conventional PR exercises, also used a medical exercise device for inspiratory muscle training (IMT) online through a mobile phone application.

In a study that used the same device for IMT, Langer et al. were the first to demonstrate that IMT with the POWERbreathe device reduced the proportion of inspiratory neural drive to the diaphragm. This has a favorable consequence for respiratory sensation and exercises tolerance, even in severe respiratory mechanical loading and tidal volume constraints [27].

The majority of the studies from the literature have a 6–12 weeks pulmonary rehabilitation design. In our country, the National Health System pays for only three weeks of hospitalized pulmonary rehabilitation. This is a major limitation on the patients and physical therapists who have to adapt to this reduced timeline.

In a study that compared online versus face-to-face PR, Bourne et al. demonstrated that online PR through a platform is non-inferior to traditional care [24]. His study duration was six weeks, and the most significant improvements were observed in the 6MWD and CAT scores. The authors exceeded the minimal important difference (MID) for the 6MWD, which is 25 m, and reduced the CAT score by 3.4 points [28]. Compared to his study, we also exceeded the MID for the 6MWD but reduced the CAT score by 4.5 points. One explanation for our findings could be that our patients used the IMT device, which reduces dyspnoea and chest tightness.

Tsai et al. also found a clinically relevant effect on 6MWD from his supervised pulmonary rehabilitation program when compared with no intervention [29]. By contrast, in a study of 22 weeks, Hansen et al. found that neither conventional PR nor supervised pulmonary telerehabilitation improved 6MWD above the MID. Explanations for his findings are that his patients had lower FEV_1_, higher symptom burden, and more exacerbations [25].

Regarding the CAT score, Hansen et al. observed that the score was statistically different at the end of the intervention, with a greater symptom reduction difference of −1.6 points in the supervised pulmonary telerehabilitation group but did not exceed the MID [25]. The minimal important difference for the CAT score is −2 points [30].

In another study, Chaplin et al. compared the effect of unsupervised web-based individual exercise and education with conventional PR and found comparable between-group effects on walking tests [25].

An interesting finding of Chaplin et al. is that although the time spent in moderate-intensity physical activity was greater in the web-based group compared to the conventional PR group, this did not translate into an increase in the total amount of moderate to vigorously physical activity. The author suggests that a more supervised approach is needed to achieve longer bouts of physical activity at the level of 3 ≥ METs [25].

In comparison to this, Loeckx et al., using a smartphone-based physical activity tele coaching approach, observed that patients requiring more contact from health care professionals experienced less physical activity benefits [31].

Compared to Loeckx’s study [32], we observed that the online group had a better improvement in their 6MWD and CAT scores compared to the inpatient group. One explanation for our findings could be that the online group could leave their homes and perform their daily living activities, thus being more active and social.

### 4.2. Strengths and Limitations

One of the current study’s strengths is that we used online IMT training using an application and thus had a better chance to improve the studied outcomes. A limitation of our study is the small number of patients and reduced days of pulmonary rehabilitation and the fact that the patients that used the application had to be connected online. Another important limitation is that we included patients who had a stationary bicycle at home for endurance training in the online PR group. Considering this factor, it would have been interesting to see what the evolution would have been for these patients if they could not perform endurance training. In the future, for those who want to perform online pulmonary rehabilitation, a pulse-oximeter, smartphone, and POWERbreathe device should be provided.

## 5. Conclusions

In conclusion, online pulmonary rehabilitation using a mobile phone application was not inferior to traditional inpatient pulmonary rehabilitation. As expected, improvements in all outcomes were found when comparing pre-test and post-test results of each of the two tests. In a direct comparison of pre-test and post-test variations, the online rehabilitation method showed better results regarding MIP (%), 6MWD (%), and CAT scores. However, the MEP (%) variation was significantly higher in patients undergoing the traditional rehabilitation method. Further studies are needed to demonstrate the utility and feasibility of mobile phone applications for pulmonary rehabilitation in patients with COPD.

## Figures and Tables

**Table 1:** Comparison of pre-test and post-test results in the inpatient setting.

Variables *	Pre-Test (n = 72)	Post-Test (n = 72)	*p*-Value
Age, years (mean ± SD)	64.9 ± 5.7	64.9 ± 5.7	-
Sex (men) **	54 (75.0%)	54 (75.0%)	-
BMI, kg/m^2^ (mean ± SD)	25.4 ± 3.3	25.4 ± 3.3	-
FVC (L) pred	4.1 ± 0.4	4.1 ± 0.4	-
FVC (L) actual	2.9 ± 0.4	3.0 ± 0.3	0.091
FVC (%)	70.8 ± 5.9	70.1 ± 5.9	0.477
FEV1 (L) pred	3.0 ± 0.3	3.0 ± 0.3	-
FEV1 (L) actual	1.4 ± 0.2	1.3 ± 0.3	0.200
FEV1 (%)	42.5 ± 4.6	43.1 ± 4.5	0.430
FEV1/FVC (%) pred	74.6 ± 1.1	74.6 ± 1.1	-
FEV1/FVC (%) actual	44.9 ± 5.7	45.2 ± 5.7	0.752
MIP (cmH2O) pred	103.3 ± 4.5	103.3 ± 4.5	-
MIP (cmH2O) actual	55.7 ± 15.8	62.5 ± 16.6	0.012
MIP (%)	53.8 ± 14.5	60.4 ± 15.1	0.008
MEP (cmH2O) pred	112.7 ± 4.6	112.7 ± 4.6	-
MEP (cmH2O) actual	82.2 ± 12.3	86.8 ± 12.5	0.027
MEP (%)	72.8 ± 9.9	76.9 ± 9.9	0.038
6MWT (m) pred	467.6 ± 35.0	467.6 ± 35.0	-
6MWT (m) actual	340.5 ± 85.0	371.5 ± 79.6	0.025
6MWT (%)	72.5 ± 15.6	79.2 ± 14.4	0.008
CAT	19.5 ± 5.1	14.7 ± 4.1	<0.001
mMRC (mean rank)	45.25	27.75	<0.001

* Data reported as mean ± SD and calculated using Student’s *t*-test; ** Data reported as n (%), and calculated using Chi-square test; SD—standard deviation; BMI—body mass index; FVC—forced vital capacity; FEV1—forced expiratory volume in the first second; MIP—maximal inspiratory pressure; MEP—maximal expiratory pressure; 6MWT—6-min walking test; CAT—COPD assessment test; mMRC—modified Medical Research Council scale.

**Table 2:** Comparison of pre-test and post-test in the online setting.

Variables *	Pre-Test (n = 58)	Post-Test (n = 58)	*p*-Value
Age, years (mean ± SD)	64.3 ± 4.3	64.3 ± 4.3	-
Sex (men) **	42 (72.4%)	42 (72.4%)	-
BMI, kg/m^2^ (mean ± SD)	25.7 ± 2.5	25.7 ± 2.5	-
FVC (L) pred	4.3 ± 0.3	4.3 ± 0.3	-
FVC (L) actual	3.0 ± 0.4	3.1 ± 0.4	0.896
FVC (%)	71.0 ± 6.8	71.4 ± 6.6	0.830
FEV1 (L) pred	3.3 ± 0.3	3.3 ± 0.3	-
FEV1 (L) actual	1.3 ± 0.2	1.4 ± 0.2	0.800
FEV1 (%)	41.7 ± 4.6	42.2 ± 4.6	0.674
FEV1/FVC (%) pred	74.8 ± 0.9	74.8 ± 0.9	-
FEV1/FVC (%) actual	44.2 ± 6.5	44.5 ± 6.2	0.894
MIP (cmH2O) pred	103.8 ± 3.4	103.8 ± 3.4	-
MIP (cmH2O) actual	55.7 ± 12.1	59.9 ± 12.3	0.194
MIP (%)	53.7 ± 11.6	62.2 ± 13.3	0.004
MEP (cmH2O) pred	113.2 ± 3.5	113.2 ± 3.5	-
MEP (cmH2O) actual	80.2 ± 13.6	83.3 ± 13.1	0.307
MEP (%)	70.9 ± 12.0	74.1 ± 11.5	0.145
6MWT (m) pred	473.0 ± 35.0	467.6 ± 35.0	-
6MWT (m) actual	342.9 ± 61.9	387.3 ± 56.3	0.006
6MWT (%)	72.5 ± 12.5	81.9 ± 11.3	<0.001
CAT	20.1 ± 5.3	13.9 ± 4.5	<0.001
mMRC (mean rank)	39.4	19.5	0.004

* Data reported as mean ± SD and calculated using Student’s *t*-test; ** Data reported as n (%) and calculated using Chi-square test; SD—standard deviation; BMI—body mass index; FVC—forced vital capacity; FEV1—forced expiratory volume in the first second; MIP—maximal inspiratory pressure; MEP—maximal expiratory pressure; 6MWT—6-min walking test; CAT—COPD assessment test; mMRC—modified Medical Research Council scale.

**Table 3:** Comparison of pre-test results between inpatients and online participants.

Variables *	Inpatient (n = 72)	Online (n = 58)	*p*-Value
Age, years (mean ± SD)	64.9 ± 5.7	64.3 ± 4.3	0.659
Sex (men) **	54 (75.0%)	42 (72.4%)	0.727
BMI, kg/m^2^ (mean ± SD)	25.4 ± 3.3	25.7 ± 2.5	0.689
FVC (L) pred	4.1 ± 0.4	4.3 ± 0.3	0.133
FVC (L) actual	2.9 ± 0.4	3.0 ± 0.4	0.105
FVC (%)	70.8 ± 5.9	71.0 ± 6.8	0.930
FEV1 (L) pred	3.0 ± 0.3	3.3 ± 0.3	0.299
FEV1 (L) actual	1.4 ± 0.2	1.3 ± 0.2	0.066
FEV1 (%)	42.5 ± 4.6	41.7 ± 4.6	0.479
FEV1/FVC (%) pred	74.6 ± 1.1	74.8 ± 0.9	0.639
FEV1/FVC (%) actual	44.9 ± 5.7	44.2 ± 6.5	0.654
MIP (cmH2O) pred	103.3 ± 4.5	103.8 ± 3.4	0.659
MIP (cmH2O) actual	55.7 ± 15.8	55.7 ± 12.1	0.978
MIP (%)	53.8 ± 14.5	53.7 ± 11.6	0.959
MEP (cmH2O) pred	112.7 ± 4.6	113.2 ± 3.5	0.662
MEP (cmH2O) actual	82.2 ± 12.3	80.2 ± 13.6	0.536
MEP (%)	72.8 ± 9.9	70.9 ± 12.0	0.482
6MWT (m) pred	467.6 ± 35.0	473.0 ± 35.0	0.496
6MWT (m) actual	340.5 ± 85.0	342.9 ± 61.9	0.899
6MWT (%)	72.5 ± 15.6	72.5 ± 12.5	0.967
CAT	19.5 ± 5.1	20.1 ± 5.3	0.608
mMRC (mean rank)	45.25	39.4	0.696

* Data reported as mean ± SD and calculated using Student’s *t*-test; ** Data reported as n (%), and calculated using Chi-square test; SD—standard deviation; BMI—body mass index; FVC—forced vital capacity; FEV1—forced expiratory volume in the first second; MIP—maximal inspiratory pressure; MEP—maximal expiratory pressure; 6MWT—6-min walking test; CAT—COPD assessment test; mMRC—modified Medical Research Council scale.

**Table 4:** Comparison of post-test results between inpatients and online participants.

Variables *	Inpatient (n = 72)	Online (n = 58)	*p*-Value
Age, years (mean ± SD)	64.9 ± 5.7	64.3 ± 4.3	0.508
Sex (men) **	54 (75.0%)	42 (72.4%)	0.727
BMI, kg/m^2^ (mean ± SD)	25.4 ± 3.3	25.7 ± 2.5	0.568
FVC (L) pred	4.1 ± 0.4	4.3 ± 0.4	0.502
FVC (L) actual	2.9 ± 0.4	3.1 ± 0.4	0.004
FVC (%)	71.3 ± 5.9	71.4 ± 6.6	0.927
FEV1 (L) pred	3.0 ± 0.3	3.2 ± 0.3	0.140
FEV1 (L) actual	1.2 ± 0.2	1.4 ± 0.2	0.010
FEV1 (%)	43.1 ± 4.5	42.2 ± 4.6	0.263
FEV1/FVC (%) pred	74.6 ± 1.2	74.8 ± 0.9	0.426
FEV1/FVC (%) actual	45.2 ± 5.7	44.5 ± 6.2	0.461
MIP (cmH2O) pred	103.3 ± 4.5	103.8 ± 3.4	0.639
MIP (cmH2O) actual	62.6 ± 16.6	59.9 ± 12.3	0.602
MIP (%)	60.4 ± 15.1	62.2 ± 13.2	0.659
MEP (cmH2O) pred	112.7 ± 4.6	113.2 ± 3.5	0.477
MEP (cmH2O) actual	86.9 ± 12.5	83.8 ± 13.1	0.614
MEP (%)	76.9 ± 9.9	74.1 ± 11.5	0.662
6MWT (m) pred	467.6 ± 35.0	473.0 ± 25.7	0.346
6MWT (m) actual	371.5 ± 79.6	387.3 ± 56.3	0.293
6MWT (%)	79.2 ± 14.4	81.9 ± 11.3	0.245
CAT	14.7 ± 4.1	13.9 ± 4.5	0.291
mMRC (mean rank)	35.33	30.10	0.222

* Data reported as mean ± SD and calculated using Student’s *t*-test; ** Data reported as n (%), and calculated using Chi-square test; SD—standard deviation; BMI—body mass index; FVC—forced vital capacity; FEV1—forced expiratory volume in the first second; MIP—maximal inspiratory pressure; MEP—maximal expiratory pressure; 6MWT—6-min walking test; CAT—COPD assessment test; mMRC—modified Medical Research Council scale.

**Table 5:** Comparison of mean differences in rehabilitation results between inpatients and online participants.

Variables *	Inpatient (n = 72)	Online (n = 58)	*p*-Value
FVC (L) actual	0.1 ± 0.1	0.1 ± 0.1	1
FVC (%)	0.7 ± 0.1	0.4 ± 0.2	<0.001
FEV1 (L) actual	0.1 ± 0.1	0.1 ± 0.1	1
FEV1 (%)	0.6 ± 0.1	0.5 ± 0.1	<0.001
FEV1/FVC (%) actual	0.3 ± 0.1	0.3 ± 0.1	1
MIP (cmH2O) actual	6.8 ± 0.1	4.2 ± 0.3	<0.001
MIP (%)	6.6 ± 0.6	8.5 ± 1.3	<0.001
MEP (cmH2O) actual	4.6 ± 0.2	3.1 ± 0.5	<0.001
MEP (%)	4.1 ± 0.1	3.2 ± 0.1	<0.001
6MWT (m) actual	31.0 ± 5.4	44.4 ± 5.6	<0.001
6MWT (%)	6.7 ± 1.2	9.4 ± 1.2	<0.001
CAT	4.8 ± 1.0	6.2 ± 0.8	<0.001
mMRC (mean rank)	17.5	19.9	<0.001

* Data reported as mean ± SD and calculated using Student’s *t*-test; SD—standard deviation; BMI—body mass index; FVC—forced vital capacity; FEV1—forced expiratory volume in the first second; MIP—maximal inspiratory pressure; MEP—maximal expiratory pressure; 6MWT—6-min walking test; CAT—COPD assessment test; mMRC—modified Medical Research Council scale.

## Data Availability

The data presented in this study are available on request from the corresponding author.
